# α-Chymotrypsin Immobilized on a Low-Density Polyethylene Surface Successfully Weakens *Escherichia coli* Biofilm Formation

**DOI:** 10.3390/ijms19124003

**Published:** 2018-12-12

**Authors:** Cristina Cattò, Francesco Secundo, Garth James, Federica Villa, Francesca Cappitelli

**Affiliations:** 1 Department of Food, Environmental and Nutritional Sciences, Università degli Studi di Milano, Milano 20133, Italy; cristina.catto@unimi.it (C.C.); federica.villa@unimi.it (F.V.); 2 Institute of Chemistry of Molecular Recognition, National Research Council, Milano 20131, Italy; francesco.secundo@icrm.cnr.it; 3 Center for Biofilm Engineering, Montana State University, Bozeman, MT 59717, USA; gjames@montana.edu

**Keywords:** biofilm, anti-biofilm surface, surface functionalization, α-chymotrypsin, proteinase

## Abstract

The protease α-chymotrypsin (α-CT) was covalently immobilized on a low-density polyethylene (LDPE) surface, providing a new non-leaching material (LDPE-α-CT) able to preserve surfaces from biofilm growth over a long working timescale. The immobilized enzyme showed a transesterification activity of 1.24 nmol/h, confirming that the immobilization protocol did not negatively affect α-CT activity. Plate count viability assays, as well as confocal laser scanner microscopy (CLSM) analysis, showed that LDPE-α-CT significantly impacts *Escherichia coli* biofilm formation by (i) reducing the number of adhered cells (−70.7 ± 5.0%); (ii) significantly affecting biofilm thickness (−81.8 ± 16.7%), roughness (−13.8 ± 2.8%), substratum coverage (−63.1 ± 1.8%), and surface to bio-volume ratio (+7.1 ± 0.2-fold); and (iii) decreasing the matrix polysaccharide bio-volume (80.2 ± 23.2%). Additionally, CLSM images showed a destabilized biofilm with many cells dispersing from it. Notably, biofilm stained for live and dead cells confirmed that the reduction in the biomass was achieved by a mechanism that did not affect bacterial viability, reducing the chances for the evolution of resistant strains.

## 1. Introduction

The main strategy for limiting bacterial loading in medical and industrial settings relies on regular cleaning and disinfection treatments aimed at killing the microbial cells on solid surfaces [1]. The incorporation of disinfectants, antiseptics, antibiotics, and metallic nanoparticles into several materials has also been proposed as a strategy to minimize pathogen growth on surfaces [2,3,4,5]. However, these strategies have shown limited efficacy and recurrent drawbacks, making their use questionable [2,6].

The major concern lies in the property of bacteria to coexist in a protective self-produced extracellular matrix within an extremely coordinated and structured surface-adhered community, known as biofilm [7]. It has been established that antibiotic overuse also triggers increased multidrug resistance in many microbial taxa [8,9]. Biofilms display high tolerance to antimicrobial agents as a result of the matrix itself, which acts as a protective barrier, and because of the reduced metabolic rate of bacteria and the presence of some dormant cells highly tolerant to a variety of drugs [10]. Additionally, most antimicrobial-releasing materials have shown a discontinuous release rate and short-term efficiency, typically no longer than 24 h, which make them less suitable for long-term applications [11,12]. 

In the last decade, researchers have concentrated their attention on approaches involving mechanisms of action that do not affect microbial life, including those that sabotage the biofilm lifestyle in a non-toxic way, and with modalities that decrease the selection pressure for drug-resistant mutations [13]. A straightforward approach is to target the biofilm matrix. Indeed, the deployment of enzymes that degrade the polymers that make up the biofilm matrix have been proposed as an effective approach to impact biofilm architecture while still preserving cell integrity [14]. Since a biofilm matrix encases bacterial cells within the biofilm colony, matrix degradation results in the destabilization of the biofilm organization and its physical integrity [15]. Therefore, the biofilm multicellular structure is compromised with a modality that does not affect cellular functions crucial for microbial survival [14]. For example, glycosidases as well as proteases or deoxyribonuclease have been reported to degrade bacterial matrix [16,17,18,19]. 

Despite these promising results, the real application of these molecules has been less feasible due to the lack of a suitable technology for efficiently retaining the anti-biofilm enzyme over a working timescale. Indeed, until now, attention has been mainly focused on the activity of enzymes in solution or as coatings. In addition, most of the effects studied occur during the initial surface attachment phase, which only partially involves matrix production by cells [13]. 

In the present work, the protease α-chymotrypsin (α-CT) was covalently immobilized on a low-density polyethylene (LDPE) surface to provide a new non-leaching material able to preserve surfaces from biofilm growth over a long working timescale. Immobilization makes enzymes more robust and more resistant to environmental changes in comparison to the counterpart free in solution [20]. More importantly, the heterogeneity of the immobilized enzyme systems allows continuous operation of enzymatic processes and rapid reactions [20]. As this strategy does not act by killing cells, it does not impose a selective pressure that would cause the onset of resistance [21]. Additionally, as the multicellularity of the biofilm is compromised, the planktonic state might be forced, restoring the efficacy of antimicrobial agents [22]. Notably, in this paper the anti-biofilm activity of immobilized enzymes was tested using a high-level and realistic approach by setting up an *Escherichia coli* laboratory-scale model system able to simulate the real conditions encountered in vivo, and provides information about the new material effect on the structure of a well-established mature biofilm [23].

## 2. Results

### 2.1. Immobilized α-CT Retains Its Activity

The possibility to link enzymes to plastics (e.g., polypropylene and polyethylene) was previously shown by our team [24]. The procedure indicates that the preventive plasma treatment of the surface is crucial for providing the plastic surface with functional groups exploitable for the covalent binding of the enzyme to the surface by glutaraldehyde (GA). Herein, on the basis of these previously reported results obtained with different proteases, α-CT immobilization was carried out by submitting LDPE coupons to a plasma treatment for 30 min and using GA as a linker. After washing (no leaching was observed on placing the coupons in water before their use) and drying, LDPE-α-CT showed transesterification activity in toluene of 1.24 nmol/h. Enzyme activity was measured in organic solvent as, in the immobilized form, the enzyme catalyzes the reaction in an insoluble form (heterogeneous solid/liquid catalysis), i.e., in the presence of mass transfer limitation conditions [25]. In this physical condition, the enzyme activity tested in organic solvent gave more reproducible results with respect to the methods carried out in aqueous media.

### 2.2. LDPE-α-CT Reduces Biofilm Biomass

**Figure 1:**
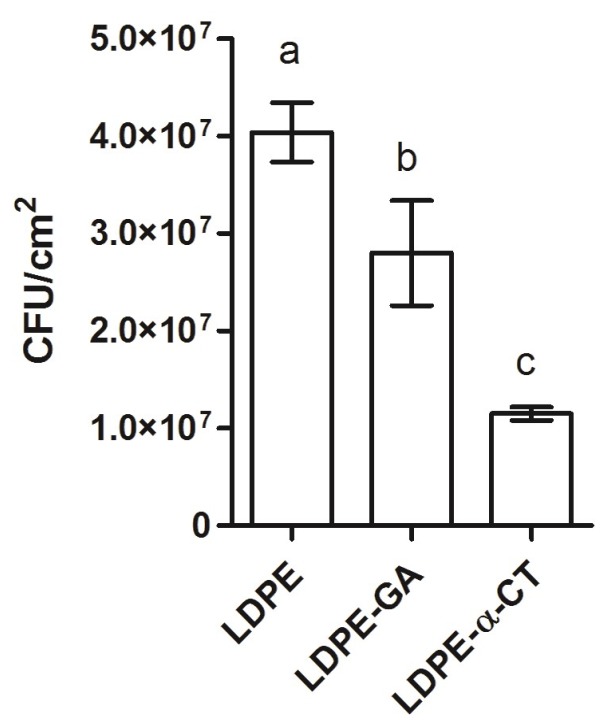
Biomass within the biofilm grown on non-functionalized (low-density polyethylene (LDPE) and LDPE-glutaraldehyde (GA)) and functionalized polyethylene surfaces (LDPE-α-chymotrypsin (α-CT)) by plate count viability assay. Data represent the mean ± standard deviation of four independent measurements. Letters a, b and c indicate significant differences (Tukey’s honest significant different (HSD) test, *p* ≤ 0.05) between the means of different surfaces.

Experiments showed that LDPE-α-CT had an optimal anti-biofilm performance, reducing viable adhered cells by 70.7 ± 5.0% in comparison to the LDPE control surface (Figure 1). Significant differences were also detected among the negative control samples, namely LDPE and LDPE-GA (Figure 1). Indeed, the number of adhered cells on LDPE-GA was 31.0 ± 5.8% lower than those attached on LDPE.

### 2.3. LDPE-α-CT Reduces Biofilm Biomass without Affecting Cell Viability

**Figure 2:**
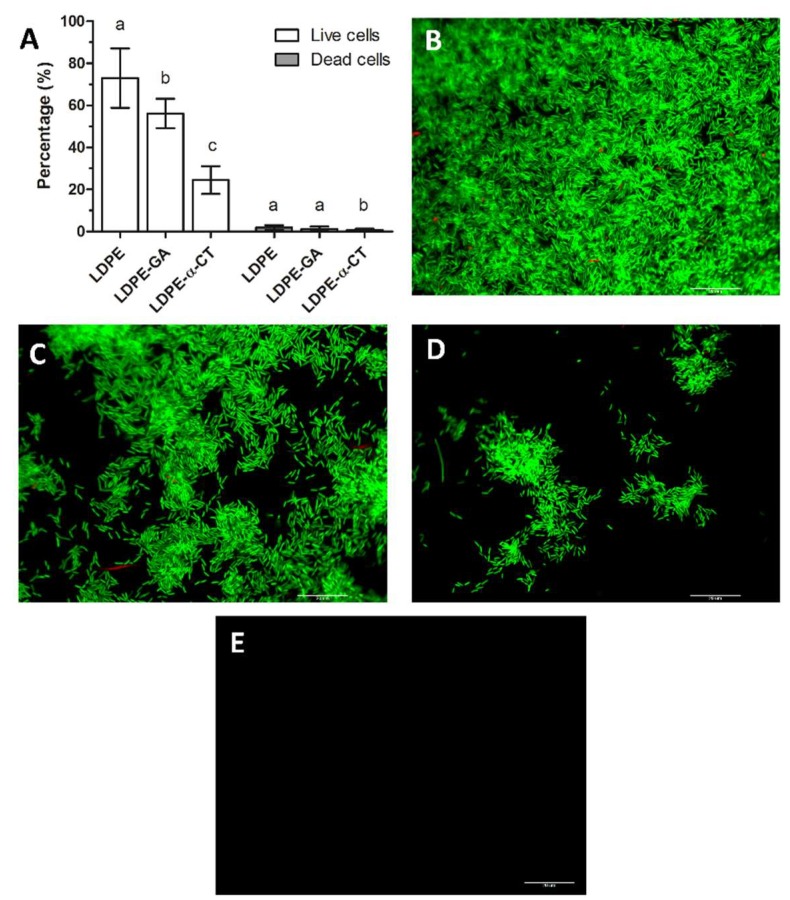
Epifluorescence microscope analysis. (**A**) Percentage of live and dead cells within the biofilm grown on non-functionalized (LDPE and LDPE-GA) and functionalized polyethylene surfaces (LDPE-α-CT). Data represent the mean ± standard deviation of four independent measurements. Letters a, b and c indicate significant differences (Tukey’s HSD, *p* ≤ 0.05) between the means of different surfaces. (**B**–**D**) Representative epifluorescence microscope images of *E. coli* biofilm stained with a Live/Dead BacLight viability kit and grown on LDPE (**B**), LDPE-GA (**C**), and LDPE-α-CT (**D**) surfaces (60×, 1.0 NA water immersion objective). Green fluorescence corresponds to *E. coli* live cells (λ_ex_: 480 nm and λ_em_: 516 nm) and red fluorescence corresponds to *E. coli* dead cells (λ_ex_: 581 nm and λ_em_: 644 nm). (**E**) Representative epifluorescence microscope image of LDPE, LDPE-GA, and LDPE-α-CT without biofilm and stained with a Live/Dead BacLight viability kit showing no detectable fluorescence. Scale bar = 20 µm.

Epifluorescence microscopic techniques were additionally used to provide image analysis and in situ quantification of bacterial cells. Figure 2 shows direct microscope visualizations of the total biofilm biomass on functionalized and non-functionalized coupons, stained for live and dead cells. 

Microscope assay revealed that the biofilm displayed a number of dead cells lower than 1.9 ± 1.1%, with no significant differences in the percentage of stained area between the LDPE and LDPE-GA surfaces, whereas a lower number of dead cells were found on LDPE-α-CT (Figure 2) (dead cells: LDPE: 1.9 ± 1.1%; LDPE-GA: 1.3 ± 1.1%; LDPE-α-CT: 0.7 ± 0.5%).

Biofilm on LDPE-α-CT showed a decreased number of live cells compared to the LDPE control surface, by up to 66.4 ± 11.0%, confirming results obtained in the plate count viability assay (Figure 2) (live cells: LDPE: 73.0 ± 14.1%; LDPE-α-CT: 24.5 ± 6.6%). A reduction of 23.9 ± 2.9% in the percentage of live cells on LDPE-GA compared to LDPE cells was also detected (live cells: LDPE-GA: 56.2 ± 7.0%) (Figure 2).

Statistical analysis of relative viability revealed no significant difference between LDPE, LDPE-GA, and LDPE-α-CT materials (relative viability: LDPE: 39.3 ± 15.5; LDPE-GA: 42.6 ± 7.5; LDPE-α-CT: 36.3 ± 4.8).

Coupons without biofilm and stained with the same dye did not produce detectable fluorescence, therefore no false positive signals were produced (Figure 2E).

### 2.4. LDPE-α-CT Affect Biofilm Morphology

**Figure 3:**
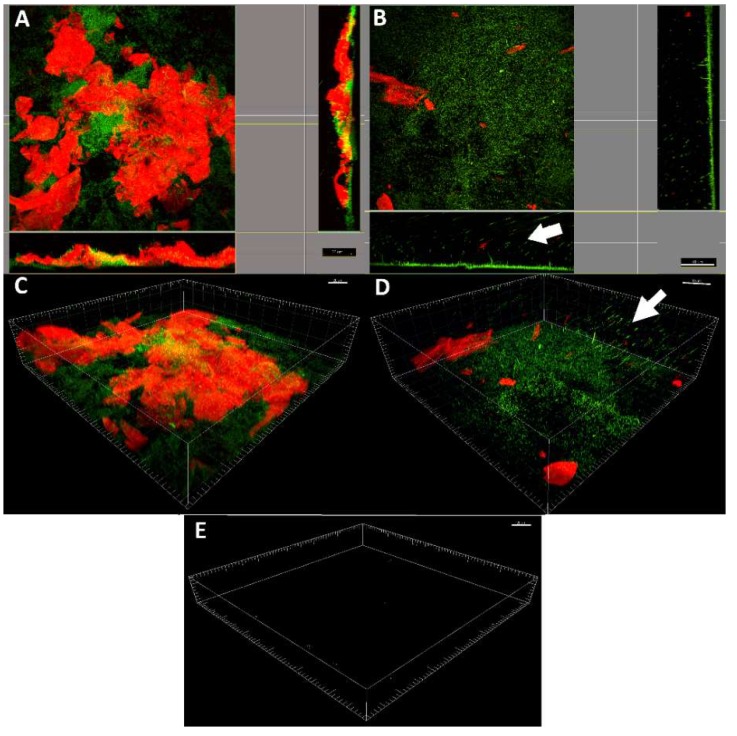
Confocal laser scanning microscopy analysis. Representative projection analysis (**A**,**B**) and three-dimensionally (3D) reconstructed CLSM images (**C**,**D**) of *E. coli* biofilm grown on non-functionalized LDPE surface (**A**,**C**) and LDPE-α-CT (**B**,**D**) functionalized surface (63×, 0.9 NA water immersion objective). The arrows indicate cells detaching from the biofilm. Live cells were stained green with SYBR Green I (λ_ex_ at 488 nm, λ_em_ at 520 nm), whereas the polysaccharide matrix was stained red with Texas Red-labeled concanavalin A (ConA) (λ_ex_ at 543 nm, λ_em_ at 615 nm). (**E**) Representative 3D reconstructed CLSM images of LDPE and LDPE-α-CT without biofilm and stained with SYBR Green I or Texas Red-labeled ConA showing no detectable fluorescence. Scale bar = 20, 30, or 40 µm.

Biofilm morphology was assessed by confocal laser scanner microscopy (CSLM) after staining with SYBR Green I and Texas Red-labeled concanavalin A. Projection analysis as well as three-dimensionally (3D) reconstructed CLSM images showed a complex biofilm on the LDPE biofilm with an intense red and green signal corresponding to multi-layers of cells (green signal) organized in macro-colonies inside a well-structured polysaccharide matrix (red signal) (Figure 3A–C). Interestingly, cells were mostly located at the bottom of the biofilm, in contact with the surface, whereas the matrix was generally found over the cellular component. On the contrary, biofilm growth on LDPE-α-CT resulted in a significant decrease of thickness with a mono-layer of dispersed cells and very low presence of polysaccharide matrix (Figure 2B–D). In addition, there were many cells dispersing from the biofilm.

No detectable fluorescence was observed when coupons without biofilm were stained with the same dyes, indicating that false positive signals were not produced (Figure 3E).

Table 1 provides the morphological parameters of biofilm grown on LDPE, LDPE-GA, and LDPE-α-CT. CLSM image analysis showed that the biofilm grown on LDPE and LDPE-GA control surfaces reached about 20 µm in thickness. On the contrary, the biofilm on LDPE-α-CT displayed a thickness below 4 µm, with a decrease of up to 81.8 ± 16.7% with respect to the biofilm grown on LDPE. Additionally, biofilm roughness was slightly decreased in both LDPE-GA (−13.8 ± 1.7%) and LDPE-α-CT (−13.8 ± 2.8%), indicating a more uniform biofilm layer in comparison to the control LDPE biofilm. On LDPE-α-CT, the substratum covered by biofilm was significantly lower (−63.1 ± 1.8%) than that in the corresponding non-functionalized LDPE. Interestingly, a small reduction in the substratum coverage was also recorded for the biofilm grown on LDPE-GA (−13.8 ± 1.7%). Total bio-volume of the biofilm grown on LDPE-α-CT was found to be significantly decreased compared to both LDPE and LDPE-GA biofilms, with a reduction of up to the 78.0 ± 16.1% in comparison to the LDPE control surface. Indeed, LDPE-α-CT exhibited a reduced cellular bio-volume by 71.7 ± 3.4% and a reduced polysaccharide matrix bio-volume by 80.2 ± 23.2% compared to the LDPE surface. Additionally, a statistically significant reduction in the cellular bio-volume was found in the biofilm grown on LDPE-GA compared to that grown on LDPE (−21.8 ± 1.0%). The matrix/cell bio-volume ratio always displayed a value over 1.9 with no statistically significant differences among surfaces, which indicates a predominance of matrix with respect to the cellular component. The biofilm exposed surface/bio-volume ratio significantly increased when biofilm was grown on LDPE-α-CT (7.1 ± 0.2-fold) whereas no significant differences were detected between biofilms grown on LDPE and LDPE-GA.

## 3. Discussion

In this study, the protease α-CT was covalently and irreversibly immobilized on an LDPE surface to provide a new material with anti-biofilm properties. LDPE was chosen as it is a polymer with excellent chemical resistance, low wetting properties in aqueous media, high impact strength, light weight, and high flexibility [26,27] and is currently used for many applications, e.g., biomedical devices [28] and food packaging [29].

Among others (e.g., subtilisin Carlsberg from *Bacillus licheniformis*, lipase from *Burkholderia cepacia* or pectinase from *Aspergillus niger*), α-CT was preferred as, in a previous work by these authors, this enzyme showed the highest transesterification activity once immobilized with GA [24,30]. Notably, the same authors observed an increase in the catalytic activity of α-CT after treatment with GA [31]. In addition, several studies confirmed the ability of α-CT to limit biofilm formation on solid surfaces, both free-in-solution treatment as well as in coatings [32,33,34,35,36,37]. 

Beside the previously promising results, the use of the free enzyme has drawbacks resulting from sensitivity to process conditions, low stability, or propensity to be inhibited by other molecules. Indeed, applications are limited by the lack of long-term operational stability and the technical challenge of enzyme recovery and reuse [38]. Additionally, enzyme coatings suffer decreased activity as degradation of the coating quickly occurs [39].

Compared to the enzyme used free in solution as well as in coatings, chemical immobilization ensures the retention of the catalytic activity, which allows the enzyme to be used repeatedly and continuously, as well as confining the protease activity where biofilm formation occurs [30,38]. Indeed, the nature of the covalent binding guarantees the long life of the material since molecules are permanently attached and integrated into the polymer scaffold structure [40], preserving the surrounding environment from enzyme contamination. This is especially useful in those fields where chemical contamination in the final product must be avoided for safety reasons, e.g., in food contact processing surfaces [41]. In addition, immobilization enhances the enzyme stability under both storage and operational conditions, e.g., by increasing its thermal stability, and therefore making it more attractive for diverse applications, especially when surfaces are subjected to harsh reaction conditions [24,31,42]. Recently, Spadoni-Andreani et al. [37] covalently linked α-CT on polypropylene, thus providing a new material able to preserve the surface from *Candida albicans* colonization. The authors carried out covalent enzyme immobilization by activating the surface with a plasma treatment and linking the enzyme with GA. Herein, a similar protocol was applied, and α-CT was immobilized on LDPE coupons after a plasma treatment of 30 min and using GA as a linker. 

Plasma technology was previously employed to improve LDPE surface properties, leading to the generation of activated species including hydrophilic functional groups on the first molecular layers of the material [40]. Functional groups allowed the initiation of the surface enzyme immobilization using GA as a linker. Among various cross-linking agents, GA has been long used for protein immobilization, including a number of enzymes [43,44,45]. Indeed, GA has been successfully used to covalently immobilize α-CT onto modified polyvinyl chloride microspheres [46] as well as on silica beads [47]. Here, for the first time, the peculiar properties of plasma technology and GA were combined, giving the right condition for α-CT immobilization on the LDPE surface. Indeed, the plasma treatment was crucial for providing the plastic surface with functional groups exploitable for the covalent binding of the enzyme to the surface, whereas the inclusion of the spacer GA was essential to improve conformational flexibility, to restrict interaction among immobilized enzyme molecules, and to enhance enzymatic activity [37]. Previous literature also highlighted the role of GA in retaining much of the original activity of enzyme when used as linker in the immobilization process, allowing the new material to be reused more than six times without loss of efficacy [46]. The combination of plasma treatment with a linker seems also to be instrumental to retain the anti-biofilm activity over a long timescale. Indeed, in the past, the coupling of plasma treatment with the linker 2-hydroxyethyl methacrylate was used to functionalize LDPE surfaces with small molecules, providing new materials able to maintain their anti-biofilm performance after having been used for more than 10 times [40].

In a previous work by these authors, it was shown that α-CT activity was negligibly affected by the immobilization reaction with GA. Furthermore, it was shown that GA has an activating effect on α-CT [31]. In line with the previous work, α-CT immobilized on LDPE showed protease activity, confirming that immobilization did not affect the enzyme activity. This promising result opens up the possibility to extend the adopted immobilization protocol to other polymeric materials. Notably, the use of plasma technology coupled with GA makes all surfaces, including those that do not possess the required chemical features, suitable for covalent binding [45,48]. Plasma sources can be also used to modify three-dimensional structures and is therefore not limited to thin, flat samples [49]. Moreover, GA is not corrosive to various substrata, including stainless steel, soft metals, rubber, and glass [50]. In this paper, the ability of the new material to affect cell adhesion and biofilm structure was evaluated against *E. coli*, using a Center for Disease Control (CDC) biofilm reactor able to simulate the conditions to which surfaces of a wide range of applications are subjected during their use, according to previous literature [51,52]. Moreover, with the aim of transferring the technology to consumer products suitable for widespread application, standard procedures were used to evaluate the efficacy of the anti-biofilm material. 

Experiments showed that the biofilm was significantly affected when grown on LDPE-α-CT. Plate count viability assays as well as CLSM analysis displayed a significant reduction in both the amount of adhered cells (over 70%) and matrix production (up to 80.2%). Biofilm stained for live and dead cells confirmed that the reduction in biomass was achieved by a mechanism that did not affect bacterial viability, reducing the chances for the evolution of resistant strains [22]. Morphology analysis displayed a statistically significant decrease of biofilm thickness on LDPE-α-CT (up to 81.8%), whereas the biofilm exposed surface/bio-volume ratio was found to be significantly increased (up to 7.1-fold). A high surface to bio-volume ratio usually corresponds to the presence of single cells and small cell clusters attached to the substratum [53]. Moreover, it is an indicative parameter of biofilm adaptation to the environment and it has been shown to increase in stress conditions [54]. Indeed, an increase in the specific surface area of the biofilm could optimize nutrient capture from the environment, especially when the role of the matrix to retain nutrient particles fails [55].

*E. coli* autotransporter adhesins, e.g., the outer membrane protein Antigen 43, were found to be instrumental in promoting cell-to-cell adhesion and aggregation at the initial stages of biofilm formation [56,57,58]. Moreover, *E. coli* proteinaceous amyloid curli fibers play important roles in the irreversible adhesion, enhance initial cell-cell interactions, and ensure the integrity of the three-dimensional biofilm architecture [59,60,61,62,63,64,65]. Notably, the inhibition of curli assembly has been found to result in a decrease of *E. coli* biofilm formation, with no apparent bactericidal or bacteriostatic effects [66]. Extracellular proteins also regulate biofilm detachment and dispersal through the enzymatic degradation of polysaccharides, proteins, and nucleic acids [67,68]. Serine proteases, including α-CT, have been reported to be effective in biofilm eradication via hydrolysis of both the proteinaceous component of the matrix and the proteins (e.g., adhesins) involved in cell adhesion to the surface [14,69,70]. Therefore, as proteins are essential for biofilm formation, their inactivation through the cleavage of their peptide bonds inevitably results in a weakened biofilm. 

According to previous literature, in this study, 3D reconstructed CLSM images showed a seriously destabilized biofilm with many cells being dispersed from the substrate. Since the extracellular matrix holds the individual cells together, the enzymatic degradation of the matrix proteins inevitably causes a massive cellular dispersal event [14,22]. Once cells have returned to the planktonic lifestyle, they are more susceptible to both immune systems and the conventional antimicrobials as compared to those in intact biofilm [71]. Additionally, the increased values of the surface to bio-volume ratio leave more biofilm surface available for antibiotic action [72]. Therefore, the combination of a LDPE-α-CT surface with conventional antimicrobial treatments might be a step toward maximizing the anti-biofilm performance of this new material, making cleaning treatments and disinfection procedures effective at low doses. This allows a more potent control against the development of drug-resistant strains [73]. 

The effect of the introduction of a GA linker into the LDPE polymer backbone on biofilm formation was also evaluated. Indeed, the experiments showed that the linked GA alone contributed, though slightly, to decrease the biofilm formation on the surface (up to 31.0%) (Figure 2A,C), with a mechanism that did not affect cell viability. A weak GA effect on *Bacillus cereus*, *Pseudomonas fluorescens*, *Staphylococcus aureus*, and *E. coli* biofilm formation was previously reported in the literature [74,75,76,77]. However, these studies highlighted that GA had a significant effect on biofilm removal, inducing sloughing events, only under long-term exposure [75]. The anti-biofilm effect of GA could be attributed to its two aldehyde groups which can interact with microbial cell constituents, among these the amino groups of the proteins within the biofilm. Indeed, GA forms methylene bridges, which may play a part in subsequent reactions including cross-linking with another protein chain in the cells. This leads to the removal of water from the biofilm followed by a dehydration effect [77,78]. In addition, it is reported that GA causes the deformation of alpha-helix structures in proteins on the outer cellular layers [79], which may include some proteins important for bacterial adhesion and biofilm formation. Indeed, studies have shown the strong binding of GA to the outer membrane proteins of *E. coli* [80], and its role in *E. coli* biofilm formation is well known [81]. Other researchers have suggested that GA also reacts with proteins of the polymeric matrix, leading to the disruption of the matrix structure [74]. Notably, GA is reported to enhance enzyme activity in the immobilization process as it introduces intermolecular cross-linking in proteins or it improves the attachment of enzyme molecules to the support [31,82].

## 4. Materials and Methods

### 4.1. Polymeric Surface Preparation

LDPE coupons (Ø 1.3 cm) were functionalized with α-CT according to Spadoni-Andreani et al. [31]. Briefly, LDPE coupons were preventively washed with bi-deionized water and acetone and then dried. Next, they were activated by exposure to O_2_ plasma for 30 min using a Harrick Plasma PDC-002 plasma cleaner (740 V, 40 mA, 29.6 W, Ithaca, NY, USA). This step is crucial for the chemical functionalization of the coupon surface (e.g., the formation of carboxyl and hydroxyl groups) [24]. Just after the plasma treatment, enzyme immobilization was carried out, loading onto the coupon 80 µL of α-CT solution (5 mg/mL) in buffer A (0.02 M potassium phosphate, pH 7.2), containing 0.005% (*v*/*v*) glutaraldehyde (GA). Then the solution was left to evaporate overnight at 25 °C and under vacuum. Control coupons were analogously prepared without α-CT. 

### 4.2. Evaluation of Immobilized α-CT Activity

Activity of the immobilized α-CT was tested in toluene (0.5 mL) measuring the alcoholysis rate of N-acetyl phenylalanine ethyl ester (1 mg/mL) in the presence of 1-propanol (5%). Tests were carried out using 3-mL vials shaken at 150 rpm at 25 °C. At a scheduled time, product formation was analyzed by a GC-FID Agilent 6850 (Santa Clara, CA, USA) networked GC system and with a polydimethylsiloxane column (30 m, 0.32 mm, film thickness 0.25 μm) (Agilent Technologies 19091Z-413E) with H_2_ as carrier gas and N_2_ and air as support gases, split 80, constant flow 2.7 mL/min, injector and FID heated at 250 °C, with a temperature ramp of 10 °C/min from 160 °C, held for 0.5 min, further heated to 240 °C, and held for 1 min.

### 4.3. E. coli Strain and Growth Condition

*E. coli* strain MG1655 was used as a model system for bacterial biofilms, being a cosmopolitan bacterium that shares a core set of genes with clinically relevant serotypes and foodborne pathogen strains, including genes involved in biofilm formation [83]. The strain was stored at −80 °C in suspensions containing 20% glycerol and 2% peptone, and was routinely grown in Luria–Bertani broth (LB, Sigma-Aldrich, St. Louis, MO, USA) at 37 °C. 

### 4.4. Biofilm Growth in the CDC Reactor

*E. coli* biofilm was grown on non-functionalized (LDPE and LDPE-GA) and functionalized (LDPE-α-CT) coupons in the CDC biofilm reactor (Biosurface Technologies, Bozeman, MT, USA) according to Cattò et al. [84]. Briefly, the bioreactor was inoculated with 400 mL of sterile LB medium with the addition of 1 mL of diluted pre-washed overnight culture containing 10^7^ cells of *E. coli*. The culture was grown at 37 °C with continuous stirring for 24 h. When the 24-h adhesion phase was over, the peristaltic pump was started and sterile 10% LB medium was continuously pumped into the reactor at a rate of 8.3 mL/min. After 48 h of dynamic phase, functionalized and non-functionalized coupons were removed, gently washed with phosphate-buffered saline (PBS, 0.01 M phosphate buffer, 0.0027 M potassium chloride, pH 7.4) and processed for analysis.

### 4.5. Plate Count Viability Assay

Collected coupons were transferred to 5 mL of PBS, and sessile cells were removed from the coupon surface by 30 s vortex mixing and 2 min sonication (Branson 3510, Branson Ultrasonic Corporation, Dunburry, CT, USA) followed by another 30 s vortex mixing. Serial dilutions of the resulting cell suspensions were plated on Tryptic Soy Agar (TSA, Fisher Scientific, Pittsburgh, PA, USA) and incubated overnight at 37 °C. Colony forming units (CFUs) were determined by the standard colony counting method. Obtained data were reported as the number of viable bacterial cells normalized to the area and means were calculated. The efficacy of the anti-biofilm material was calculated as the percentage reduction of the CFUs with respect to the LDPE control. Four coupons for each surface were analyzed. The experiment was repeated four times for a total of 16 coupons analyzed.

### 4.6. Epifluorescence Microscopy Analysis

The percentage of live and dead cells in the biofilm biomass grown on both non-functionalized and functionalized coupons was determined using a Live/Dead BacLight viability kit (L7012, Molecular Probes-Life Technologies, Thermo Fisher Scientific, Waltham, MA, USA). Biofilm was incubated with 2 µL of each fluorescent probe per ml of sterile filtered PBS at room temperature in the dark for 25 min and then rinsed with sterile PBS, according to the manufacturer’s instruction. Coupons without biofilm were also stained with the dyes in order to exclude any false positive signals. Biofilm samples were visualized using a Nikon Eclipse E800 epifluorescent microscope with excitation at 480 nm and emission at 516 nm for the green channel and excitation at 581 nm and emission at 644 nm for the red channel (Tokyo, Japan). Images were captured with a 60×, 1.0 NA water immersion objective and analyzed via MetaMorph 7.5 software (Molecular Devices, Sunnyvale, CA, USA). The percent area of stained cells was obtained by calculating at least 10 random images for each sample. The efficacy of the anti-biofilm material was calculated as the percentage reduction in the stained cell area with respect to the LDPE control images. Relative viability within the biofilm was determined by dividing the percent area of the live cells by the percent area of the dead cells in each sample. Four coupons of each surface were analyzed. The experiment was repeated four times for a total of 16 coupons analyzed. 

### 4.7. Confocal Laser Scanning Microscopy (CLSM) Analysis

Three-dimensional morphology of biofilm growth on non-functionalized and functionalized surfaces was analyzed by CLSM according to Cattò et al. [85]. Biofilm was stained with 200 µg/mL lectin concanavalin A-Texas Red conjugate dye (C825, Molecular Probes-Life Technologies, Thermo Fisher Scientific) to visualize the polysaccharide component of the extracellular polymeric substances (EPS) and 1:1000 SYBR Green I fluorescent nucleic acid dye (S7563, Molecular Probes-Life Technologies, Thermo Fisher Scientific) to display biofilm cells, in the dark for 30 min. Coupons without biofilm were also stained in order to exclude any false positive signals. Biofilm samples were visualized using a Leica SP5 CLSM with excitation at 488 nm and emission at 520 nm for the green channel and excitation at 543 nm and emission at 615 nm for the red channel. Images were captured with a 63×, 0.9 NA water immersion objective and projections and 3D reconstructed images of biofilm were generated using the Imaris software package (Bitplane Scientific Software, Zurich, Switzerland).

Quantitative biofilm structural parameters were calculated, including (i) mean thickness, which identifies the mean distance from the substratum in the direction normal to the substrate where there is biofilm; (ii) roughness, a quantity calculated from the thickness distribution and that describes the heterogeneity of the biofilm; (iii) substratum coverage, the percentage of substrate area occupied by the biofilm; (iv) surface-to-volume ratio, which reflects the fraction of biofilm area that is exposed to nutrients; and (v) bio-volume, of both cells and the polysaccharide matrix, which provides an estimation of the biomass in the biofilm [86]. Biofilm morphological parameters were obtained via MetaMorph 7.5 (Molecular Devices, Sunnyvale, CA, USA) and COMSTAT software from at least five random images for each sample according to Heydorn et al. [53]. Four coupons of each surface were analyzed. The experiment was repeated four times for a total of 16 coupons analyzed.

### 4.8. Statistical Analysis

Two-tailed ANOVA analysis, via software run in a MATLAB environment (Version 7.0, The MathWorks Inc., Natick, MA, USA), was applied to statistically evaluate any significant differences among the samples and concentrations. The ANOVA analysis was carried out after verifying data independence (Pearson’s Chi-square test), normal distribution (D’Agostino-Pearson normality test), and homogeneity of variance (Bartlett’s test). Tukey’s honest significant different test (HSD) was used for pairwise comparison to determine the significance of the data. Statistically significant results were depicted by *p* < 0.05.

## 5. Conclusions

In this work, α-CT was successfully immobilized on an LDPE surface to provide a new material able to inhibit biofilm colonization. The multiple-target nature of the protease activity allows the new material to be used with a broad-spectrum activity against polymicrobial infections, including drug-resistant strains. Indeed, the use of drugs that impact multiple targets simultaneously is better at controlling complex disease systems, e.g., biofilms, and makes resistance development rather unlikely [87,88,89].

LDPE-α-CT may provide a solution to potentiate the anti-biofilm activity of conventional antimicrobials that are otherwise largely effective only against planktonic bacteria. Indeed, in many industrial and clinical sector surface treatments that retard biofilm formation could represent a great step forward against the challenge of biofilm formation [90]. Nowadays, the combination of antibiotics with anti-biofilm mechanisms leading to synergism is considered the best solution for the treatment of biofilm [91,92]. 

Both LDPE and α-CT are currently available at affordable prices, providing a positive foundation for the production of LDPE-α-CT at the industrial level at affordable cost. Additionally, the simple protocol for enzyme immobilization makes it suitable for application to other surfaces and complementary enzymes, e.g., those attacking other components of the biofilm matrix.

## Figures and Tables

**Table 1:** Biofilm morphological parameters of biofilm grown on non-functionalized (LDPE, LDPE-GA) and functionalized polyethylene surfaces (LDPE-α-CT). In the brackets, percentage reduction/increase in comparison to the LDPE control sample is reported when significant. Data represent the mean ± standard deviation of four independent measurements.

Parameter	LDPE	LDPE-GA	LDPE-α-CT
Thickness (µm)	20.5 ± 5.0 ^a^	19.1 ± 4.9 ^a^	3.7 ± 1.1 ^b^ (−81.8 ± 16.7)
Roughness	0.25 ± 0.03 ^a^	0.22 ± 0.02 ^b^ (−11.1 ± 1.0)	0.21 ± 0.04 ^b^ (−13.8 ± 2.8)
Substratum coverage (%)	72.3 ± 3.8 ^a^	62.3 ± 1.2 ^b^ (−13.8 ± 1.7)	26.7 ± 1.3 ^c^ (−63.1 ± 1.8)
Total bio-volume (µm^3^ µm^−2^)	89.4 ± 20.1 ^a^	76.3 ± 21.2 ^a^	19.7 ± 4.1 ^b^ (−78.0 ± 16.1)
Cells bio-volume (µm^3^ µm^−2^)	23.7 ± 4.0 ^a^	18.5 ± 0.9 ^b^ (−21.8 ± 1.0)	6.7 ± 0.3 ^c^ (−71.7 ± 3.4)
Polysaccharide matrix bio-volume (µm^3^ µm^−2^)	65.8 ± 7.8 ^a^	57.8 ± 14.9 ^a^	13.0 ± 3.8 ^b^ (−80.2 ± 23.2)
Matrix/cells bio-volume ratio	2.8 ± 0.6 ^a^	3.1 ± 0.8 ^a^	1.9 ± 0.5 ^a^
Surface/bio-volume (µm^2^ µm^−3^) × 10^−2^	1.1 ± 0.3 ^a^	1.3 ± 0.4 ^a^	8.7 ± 1.7 ^b^ (+7.8 ± 1.6-fold)

Superscript letters a, b and c indicate significant differences (Tukey’s HSD, *p* ≤ 0.05) between the means of different surfaces.
